# Early detection of transformation to BPDCN in a patient with MDS

**DOI:** 10.1186/s40164-018-0117-6

**Published:** 2018-10-06

**Authors:** Kamal Chamoun, Sanam Loghavi, Naveen Pemmaraju, Marina Konopleva, Michael Kroll, Madeleine Nguyen-Cao, Marisa Hornbaker, Courtney D. DiNardo, Tapan Kadia, Jeffrey Jorgensen, Michael Andreeff, Shimin Hu, Christopher B. Benton

**Affiliations:** 1 Departments of Leukemia, The University of Texas MD Anderson Cancer Center, 1515 Holcombe Blvd, Unit 428, Houston, TX 77030 USA; 2 Departments of Hematopathology, The University of Texas MD Anderson Cancer Center, Houston, USA; 3 Departments of Benign Hematology, The University of Texas MD Anderson Cancer Center, Houston, USA

**Keywords:** Myelodysplastic syndromes, Prognosis, BPDCN, MDS, Early detection

## Abstract

**Background:**

Blastic plasmacytoid dendritic cell neoplasm (BPDCN) is a rare and aggressive hematologic malignancy characterized by neoplastic cells that are positive for CD123, CD4, BDCA2, and TCL1 and aberrant expression of CD56. Historically, patients with BPDCN have an unfavorable prognosis and the optimal treatment is not established due to lack of prospective data.

**Case report:**

In this report we describe a patient with Felty’s syndrome and myelodysplastic syndrome (MDS) in whom a population of aberrant plasmacytoid dendritic cells emerged while on treatment with decitabine. Approximately 4 months later he transformed to leukemic BPDCN with skin and eye manifestations. Cytogenetic analysis showed diploid karyotype and molecular analysis showed mutations in KRAS, NOTCH1, and RUNX1 genes. He was treated with CD123-targeted therapy and had significant response in his marrow, skin, eyes, and functional status after one cycle.

**Conclusion:**

The case demonstrates that minimal transformative disease of BPDCN may be detectable in patients with MDS well before fulminant progression. Early detection of emerging leukemic clones may allow for alternative monitoring and treatment considerations.

## Introduction

Blastic plasmacytoid dendritic cell neoplasm (BPDCN) is a rare, aggressive hematologic malignancy that was first described in 1995 and manifests mainly as cutaneous lesions with or without lymph node or bone marrow (BM) involvement [1, 2]. BPDCN is characterized by the proliferation of intermediate-sized, monotonous, immature-appearing cells with finely dispersed chromatin and small nucleoli. The neoplastic cells are positive for CD123, CD4, BDCA2, and TCL1 and frequently show aberrant expression of CD56; they are negative for CD34 and lineage-defining markers including CD19, CD20, and myeloperoxidase (MPO) [3–5]. BPDCN affects 3 times more men than women, and is primarily a disease of elderly patients [6]. Historically, patients with BPDCN have an unfavorable prognosis, and the optimal treatment is still not established due to the lack of prospective data. Recent trials targeting the interleukin-3 (IL-3) receptor (CD123) showed promising results [7]. Association of BPDCN with MDS has been reported, yet its association with Felty syndrome has never been described [5, 8]. The current case describes a patient with Felty syndrome and MDS transformed to BPDCN with leukemic, skin, and eye manifestations. Notably, flow cytometry from longitudinal BM examinations demonstrated the emergence of an aberrant subpopulation of plasmacytoid dendritic cells well in advance of the diagnosis of BPDCN.

## Case report

A 76-year-old man presented initially for evaluation of refractory anemia. His history included a diagnosis of Felty syndrome at the age of 64 years, after he was found to have rheumatoid arthritis, neutropenia, and an enlarged spleen. A diagnosis of anemia of chronic disease was made after complete workup including a BM examination was negative for malignancy. He received multiple therapeutic agents over time to control his rheumatoid arthritis, including adalimumab, methotrexate, abatacept, infliximab, azathioprine, rituximab, and prednisone. He underwent splenectomy at 67 years.

His anemia progressed and he became transfusion dependent at the age of 68 years. He was diagnosed with myelodysplastic syndrome (MDS) after a repeat BM examination revealed minimal morphologic dysplasia with a diploid karyotype. He received erythropoietin support for 1 year with worsening of his anemia. He was next treated with adjusted-dose lenalidomide (5 mg every other day) and rapidly achieved transfusion independence and a hemoglobin level of 13 g/dL. He progressively lost his response and became transfusion dependent. After 5 years, lenalidomide was discontinued and he received one cycle of azacitidine. Azacitidine was discontinued after BM examination at that time showed no morphologic support for residual MDS, and he continued to receive transfusions for nearly one year before presenting to our institution.

**Fig. 1:**
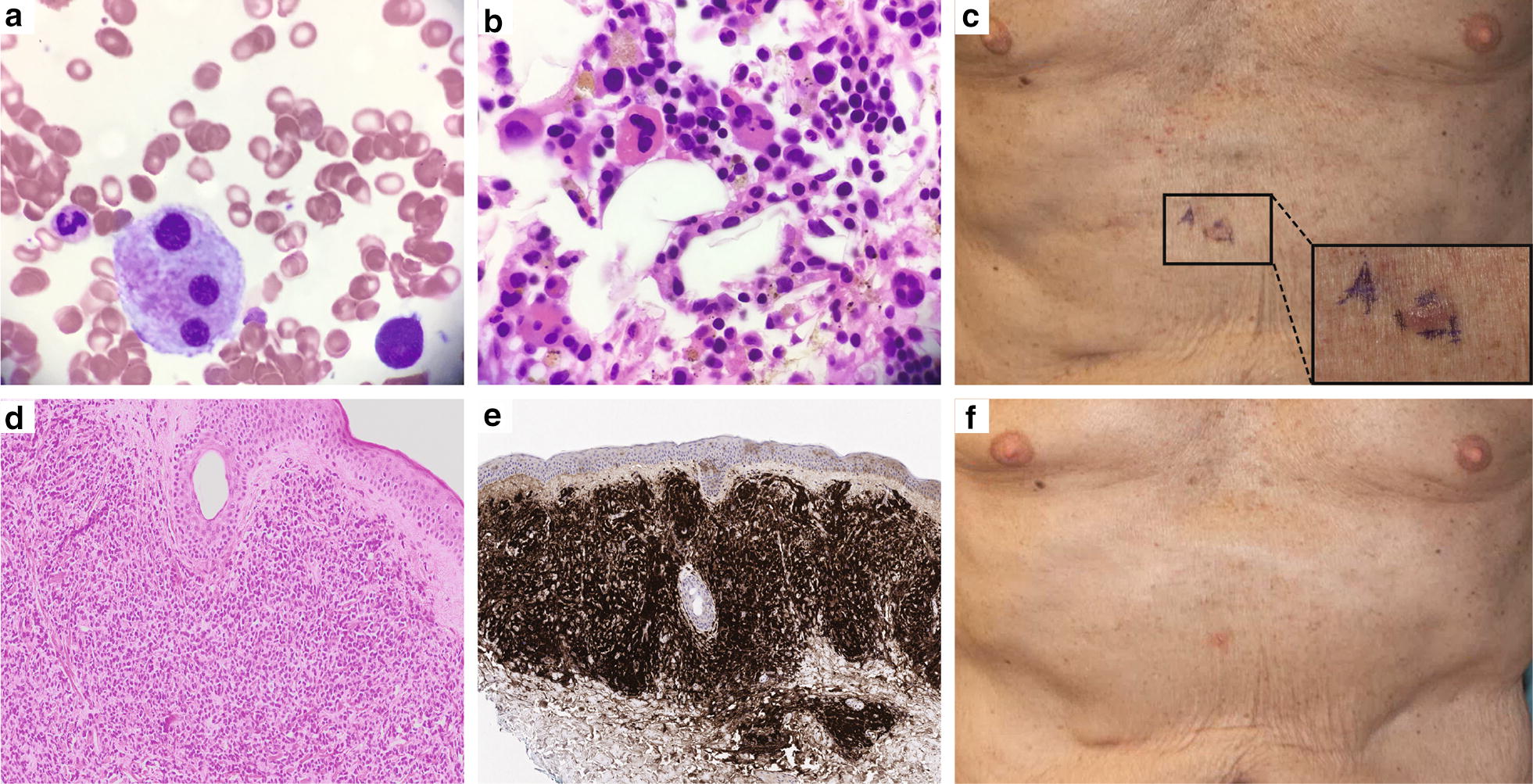
Bone marrow at presentation (MDS) and skin lesions after transformation (BPDCN). Photomicrographs of bone marrow core biopsy specimens at presentation show **a** erythroid-predominant trilineage hematopoiesis and dysplastic megakaryopoiesis with an increased number of small monolobated megakaryocytes (H&E ×400), and **b** megakaryocytes with widely separated nuclear lobes (Giemsa, ×1000). **c** Photographs show violaceous plaques on the abdomen at BPDCN diagnosis, including a truncal skin lesion on the central abdomen demarcated with blue ink, enlarged for inset. Photomicrographs of skin biopsy of the abdominal lesion showing **d** extensive dermal infiltrate of immature-appearing mononuclear cells (H&E ×40), and **e** infiltrate that is uniformly and strongly positive for CD123 (immunohistochemistry with hematoxylin counterstain, ×20). **f** Photograph of same abdominal area showing resolving lesions after 1 month of CD123-targeted therapy with SL-401

**Fig. 2:**
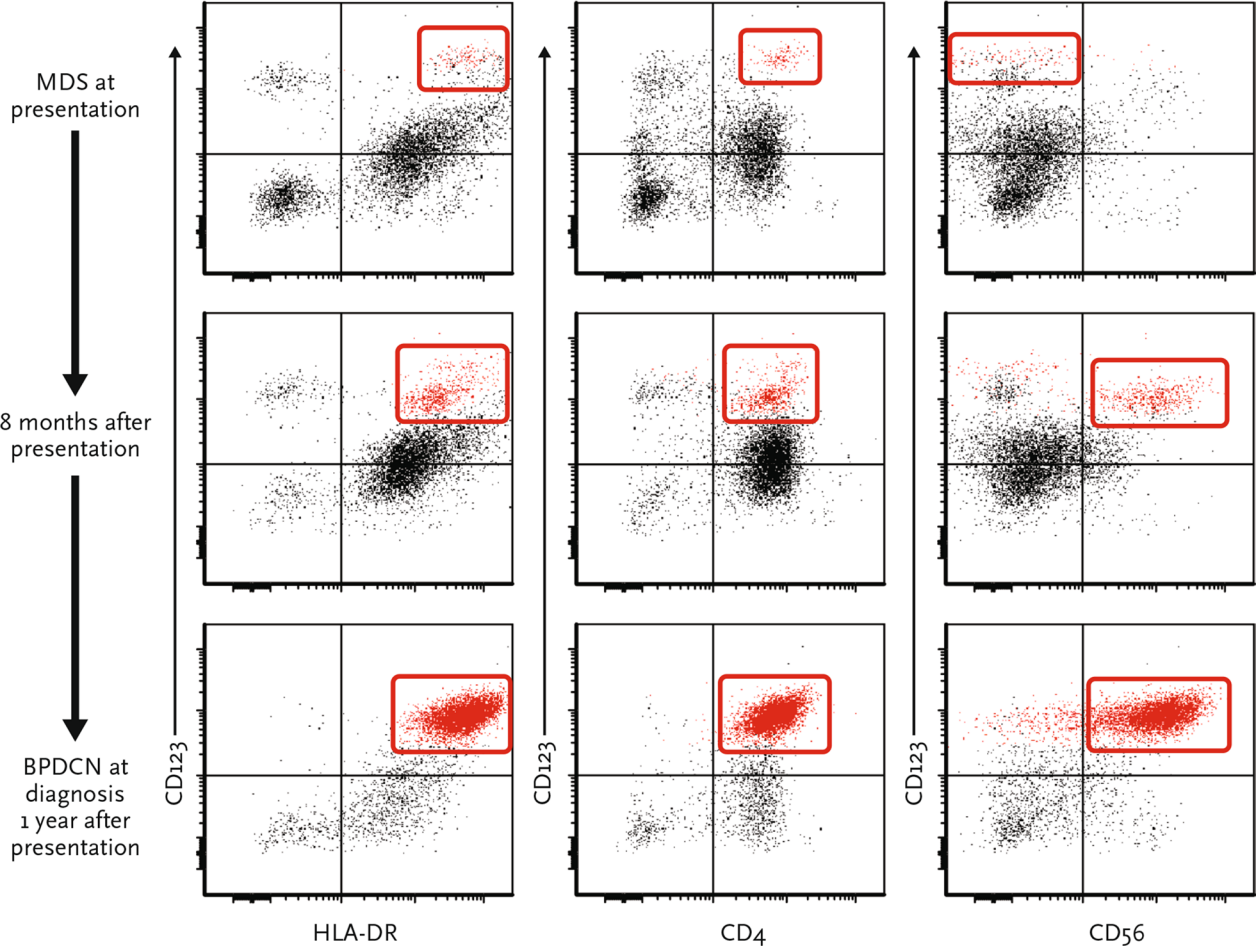
Emergence of aberrant CD123+ CD56+ CD4+ and HLA-DR + cells during the treatment period for MDS. Flow cytometry dot plots are shown for 3 longitudinal bone marrow aspirate samples: at presentation (top panel), after six courses of oral decitabine (middle panel), and at the time of fully developed BPDCN (bottom panel). The y-axis for each plot shows CD123 expression, and the x-axis shows HLA-DR, CD4 and CD56 from left to right, respectively. At presentation, the majority of PDCs had an unremarkable immunophenotype (CD123^bright^/HLA-DR^+^/CD4^+^/CD56^−^). In the interim marrow, there is the emergence of a minor, but more prominent population of aberrant PDCs that are CD56^+^ (0.79%). At diagnosis, a large population of CD123^+^ CD4^+^ CD56^+^ cells has become clearly evident

A BM core biopsy and aspiration on initial evaluation demonstrated hypercellular marrow with trilineage dysplasia, moderate reticulin fibrosis, and 2% blasts (Fig. 1a, b). Flow cytometry demonstrated changes consistent with MDS, including markedly decreased side scatter in granulocytes (cytoplasmic hypogranularity), absence of hematogones, and a small number of aberrant myeloid blasts that had expression pattern of increased CD13 and CD34 and decreased CD38. Dendritic cells with a CD123^bright^, CD4^+^, HLA-DR^+^, CD56^−^ immunophenotype were 0.2% of all analyzed cells (Fig. 2, top panel). Cytogenetic studies showed a normal diploid karyotype, and no mutations were identified on next-generation sequencing of 28 leukemia-associated genes. He was treated on clinical protocol with oral decitabine and had decreased transfusion requirements. Repeat BM aspirations showed persistence of dysplastic features and no significant increase in blasts.

Flow cytometry of a surveillance BM aspirate sample approximately 8 months after decitabine initiation showed the emergence of a minimal population of aberrant plasmacytoid dendritic cells (CD123^bright^, CD4^+^, HLA-DR^+^, CD56^+^), comprising 0.79% of cells (Fig. 2, middle panel). This represented an immunophenotypic shift from CD56^−^ plasmacytoid dendritic cells cells at presentation. After 1 year of decitabine treatment, he experienced increasing fatigue and developed scattered violaceous plaques on his abdomen, upper chest, and forearms (Fig. 1c) in addition to bilateral discoloration of the inferior conjunctiva. BM aspiration and biopsy revealed 70% immature appearing cells with finely dispersed nuclear chromatin, conspicuous nucleoli, and scant, eccentrically placed cytoplasm imparting a “hand-mirror” appearance. Immunohistochemical studies showed the immature cells were positive for CD4, CD56, CD123, and TCL1, and negative for CD34, MPO, and lysozyme. Flow cytometry showed a distinct population of CD45^dim^ cells with CD123^bright^ expression. These cells were CD4^+^, HLA-DR^+^, CD56^+^, CD34^−^, CD117^−^, CD64^−^, CD14^−^, and made up 35% of analyzed events (Fig. 2, bottom panel). This constellation of findings was diagnostic for BPDCN with extensive BM involvement. Cytogenetic studies showed a normal diploid karyotype and next-generation sequencing studies identified mutations in *KRAS*, *NOTCH1*, and *RUNX1*. Biopsy of an abdominal wall lesion showed BPDCN skin involvement (Fig. 1d, e). He was enrolled in a clinical trial (NCT02113982) of single-agent SL-401, a recombinant human IL-3 conjugated to truncated diphtheria alpha-toxin, which targets CD123. After 1 cycle, he experienced a decrease in BM blasts to 22%, resolution of skin lesions (Fig. 1f) and ophthalmic lesions, and marked improvement in performance status. After the second cycle, his BM aspirate showed 2.5% residual aberrant blastic plasmacytoid dendritic cells.

## Discussion

No association between BPDCN with Felty syndrome has previously been reported. BPDCN typically presents with asymptomatic skin nodules, patchy plaques, or bruise-like areas of discoloration. These lesions vary from < 1 to 10 cm and can be associated with erythema, hyperpigmentation, purpura, and ulceration. BM involvement is common (60–90% of patients); however, fulminant leukemia at presentation is less frequent (5–25%) [6]. Lymphadenopathy (40–50%) and splenomegaly (20%) may also be present. Extracutaneous and extramedullary manifestations are rare and include central nervous system, eye, lung, paranasal cavity, soft tissue, tonsil, and liver involvement [5]. A previous history of hematologic malignancy such as MDS, chronic myeloid leukemia, chronic myelomonocytic leukemia, or acute myeloid leukemia is present in about 10–20% of patients (MDS in < 10%) [8].

The diagnosis of BPDCN is based on the characteristic morphologic and immunophenotypic findings (expressing CD123, BDCA2, TCL1, CD4, and sometimes CD56; lack of lineage-defining markers) [3, 4, 9]. Garnache-Ottou et al. proposed a diagnostic algorithm in which all neoplasms expressing CD4 and not CD11c, MPO, cytoplasmic CD3, or cytoplasmic CD79a were to be evaluated for CD123 expression regardless of CD56 expression [4]. In addition, a report from the French Study Group on Cutaneous Lymphomas of 91 patients with BPDCN suggested that the presence of only 4 of the 5 most characteristic markers (CD123, CD4, CD56, BDCA2, and TCL1) is sufficient for the diagnosis of BPDCN [9]. Our patient had the typical immunophenotype with expression of CD123, CD4, CD56, and TCL1 and absent expression of CD34, MPO, and lysozyme. Notably, a harbinger population of aberrant plasmacytoid dendritic cells became more prominent approximately 4 months before transformation from MDS to bona fide BPDCN. This finding raises the question of whether early detection of secondary BPDCN (and other acute leukemias) might be possible by evaluating for the presence of minimal transformative disease using multiparametric flow cytometry. Such an approach might be relevant for many groups of patients, from those with high-risk MDS to those with clonal hematopoiesis of indeterminate potential.

While most patients with BPDCN have a complex karyotype, no specific chromosomal abnormality was identified in our patient. The most frequently reported abnormalities include del(5q), del(12p), del(13q), del(6q), del(15q), and del(9) [10]. *TET2* and *TP53* mutations [11], *FLT3*-internal tandem duplications [8], and *FLT3*, *RUNX2*, and *HES6* overexpression all have been reported in BPDCN patients [12]. Historically, BPDCN is associated with a median overall survival of 12–16 months [5]. For patients with isolated skin disease, treatment options may include surgical excision and radiation; however, relapses are frequent, and systemic approaches are recommended [5]. A retrospective analysis of 43 patients with leukemic presentation treated with induction chemotherapy showed a significantly higher complete remission rate in patients treated with an acute lymphocytic leukemia-type chemotherapy regimen over an acute myeloid leukemia-type [8]. Moreover, 9 of 10 BPDCN patients treated with hyper-CVAD at our institution experienced complete remission [13]. Despite initial responses, most patients relapse. Allogeneic stem cell transplant during the first complete remission may achieve durable disease control [14]. In a recent phase 1/2 trial, SL-401 achieved major responses in 7 of 9 evaluable patients with BPDCN [7]. In our patient, SL-401 as the initial BPDCN therapy achieved significant improvement in his BM, skin, eyes, and functional status after one cycle. BPDCN remains a challenging disease to manage, and additional prospective clinical trials will explore new strategies to improve clinical outcomes of patients with this aggressive disease.

Better detection and elimination of minimal residual disease after treatment of acute leukemia may offer improvements in outcomes for patients by preventing relapse [15]. Likewise, the case presented illustrates that detection of minimal transformative disease by flow cytometry may pre-date the onset of acute leukemia, and aberrant subpopulations can yield information about impending transformation. Alternative monitoring, preventive measures, and innovative therapeutic strategies at earlier stages are conceivable in patients with BPDCN.

## Abbreviations

BM: bone marrow
BPDCN: blastic plasmacytoid dendritic cell neoplasm
MDS: myelodysplastic syndrome
MPO: myeloperoxidase
IL-3: interleukin-3
